# Large Mandibular Bone Defect Reconstruction with Calcium Phosphate Cement: A Case Report

**DOI:** 10.3390/healthcare14213819

**Published:** 2026-11-09

**Authors:** Katarína Vdoviaková, Ján Danko, Lenka Krešáková, Filip Humeník, Andrej Jenča, Andrej Jenča, Janka Jenčová, Adriana Petrášová, Ľubomír Medvecký, Mária Giretová

**Affiliations:** 1 Department of Morphological Disciplines, University of Veterinary Medicine and Pharmacy in Košice, Komenského 73, 041 81 Košice, Slovakia; 2 Solmea s.r.o., Bačíkova 7, 040 01 Košice, Slovakia; 3 Division of Functional and Hybrid Materials, Institute of Materials Research of SAS, Watsonova 47, 040 01 Košice, Slovakia

**Keywords:** biocement paste, bone regeneration, biomaterial, radicular cyst, biocompatibility, dental surgery, regenerative medicine

## Abstract

**Background:** Large mandibular defects following odontogenic cyst enucleation pose reconstructive challenges because spontaneous healing is unpredictable. Calcium phosphate cements are biocompatible, osteoconductive biomaterials that may facilitate bone repair. **Case Description**: We describe reconstruction of an anterior mandibular defect in a 71-year-old woman following radicular cyst enucleation using injectable calcium phosphate cement. Preoperative assessment included clinical examination, panoramic radiography, cone-beam computed tomography (CBCT), and pulp sensibility testing. Imaging revealed a 27 × 35 × 6 mm defect. Cement was placed in the selected portion of the defect. Histopathological examination confirmed a radicular cyst; cyst contents underwent microbiological culture and antimicrobial susceptibility testing. Serial panoramic radiographs and final CBCT in August 2024 allowed assessment of radiopacity, trabecular architecture, and mandibular continuity. Throughout follow-up, seven adjacent teeth (FDI 43, 42, 41, 31, 33, 34, and 35) retained positive responses to cold and electric pulp testing, whereas tooth 32 remained nonresponsive and received no endodontic treatment. Postoperative recovery was uneventful, with no infection, biomaterial-related complications, or radiographic evidence of cyst recurrence. Follow-up imaging revealed diminishing radiographic contrast between the reconstructed region and surrounding bone, emergence of a trabecular-like pattern, and preserved mandibular continuity. These findings were consistent with healing and remodeling; however, imaging could not establish cement degradation or replacement by newly formed bone. **Conclusions:** This case demonstrates favorable clinical and radiographic outcomes following mandibular reconstruction with injectable calcium phosphate cement. Histological confirmation would be necessary to determine the extent of cement degradation and replacement by newly formed bone.

## 1. Introduction

Functional reconstruction of extensive mandibular bone defects while preserving the natural dentition remains a major challenge in contemporary oral and maxillofacial surgery [1]. Although surgical enucleation is an established treatment for benign jaw cysts, the resulting osseous defect may compromise mandibular structural integrity, periodontal support, local vascular supply, and the pulpal status of adjacent teeth [2].

Therefore, treatment success should be evaluated not only in terms of complete lesion removal but also with regard to the restoration of functional bone architecture, preservation of periodontal support, maintenance of positive pulp sensibility responses, and long-term function of the affected dentition [3,4].

Over the past four decades, calcium phosphate cements have evolved from conventional osteoconductive bone fillers into sophisticated biomaterials with enhanced biological and physicochemical properties. Their chemical composition closely resembles the mineral phase of native bone, providing a favorable environment for osteoconduction and subsequent bone remodeling [5,6]. Advances in cement formulation have improved injectability, setting characteristics, cohesion, and compressive strength, thereby facilitating surgical handling and expanding the potential applications of calcium phosphate cements (CPCs) in craniofacial reconstruction [7]. Furthermore, the incorporation of bioactive components and drug-delivery systems has extended their potential functionality beyond structural defect filling, creating opportunities for more targeted approaches to bone tissue regeneration [8,9]. Collectively, these properties make CPCs promising candidates for the reconstruction of osseous defects requiring close adaptation to surrounding tissues and adequate mechanical support while preserving the adjacent dentition [10].

The calcium phosphate cement used in the present case was selected on the basis of its physicochemical characteristics and biological properties documented in previous experimental studies. Calcium phosphate-based biomaterials have demonstrated favorable biocompatibility and osteoconductive properties, as well as the potential to support bone healing and remodeling under experimental conditions [7,11]. Their biological performance depends on several factors, including material composition, degradation kinetics, and interactions with the surrounding tissues. In particular, the release of calcium and phosphate ions may influence cellular processes associated with bone mineralization and osteogenic activity [12,13,14].

The degradation characteristics of calcium phosphate cements, particularly the associated release of calcium and phosphate ions, may influence their interactions with the surrounding bone tissue [11,15]. Experimental studies suggest that these processes may contribute to osteogenic cellular activity and mineral deposition, thereby potentially supporting bone healing and remodeling [16,17]. However, neither cement degradation nor the underlying cellular mechanisms were directly investigated in the present clinical case.

Previous experimental investigations have examined the physicochemical characteristics, biocompatibility, and regenerative potential of calcium phosphate-based biocements in both in vitro and in vivo settings. Medvecký et al. [18] characterized calcium phosphate-based cement formulations and evaluated their performance through in vitro analyses and in vivo experiments. Subsequent preclinical studies investigated the biological responses to these materials and their modified formulations in animal models. Tóthová et al. [19] examined changes in selected biochemical and inflammatory markers following the treatment of experimentally induced cartilage defects in sheep, whereas Krešáková et al. [20] and Vdoviaková et al. [21] evaluated tissue repair and healing outcomes in ovine and porcine models, respectively. Varga et al. [22] further investigated a modified tetracalcium phosphate-based biocement for osteochondral defect repair in sheep. Biocement C constitutes a foundational calcium phosphate cement formulation from which subsequent modified biomaterials have been developed. Although these investigations provide relevant preclinical evidence regarding the biological performance of calcium phosphate-based cements, their findings cannot be directly extrapolated to the clinical application described in the present report. Nevertheless, they establish a scientific rationale for investigating Biocement C as a potential reconstructive material for mandibular bone defects.

Despite the growing interest in calcium phosphate-based biomaterials for craniofacial reconstruction, clinical evidence supporting their application in extensive mandibular defects following cyst enucleation remains limited. Existing studies have primarily focused on radiographic outcomes and biomaterial integration, whereas the preservation of pulp sensibility responses and long-term function of adjacent teeth has received comparatively little attention [23]. Consequently, well-documented clinical cases addressing both the radiographic evolution of reconstructed mandibular defects and the preservation of the natural dentition are particularly valuable for informing future clinical investigations.

This case report describes the reconstruction of an extensive anterior mandibular defect following radicular cyst enucleation in a 71-year-old woman. Calcium phosphate cement was applied to a surgically selected portion of the resulting osseous defect, which measured 27 × 35 × 6 mm. The report documents the clinical course and long-term radiographic findings, including changes consistent with progressive bone healing and remodeling, preservation of mandibular continuity, and sustained positive pulp sensibility responses in seven adjacent teeth. By presenting these observations, this case contributes to the limited clinical literature on calcium phosphate cement application in extensive mandibular defect reconstruction and underscores the need for further systematic clinical investigation [23].

## 2. Case Report

### 2.1. Patient Presentation, Clinical and Radiographic Examination

**Figure 1:**
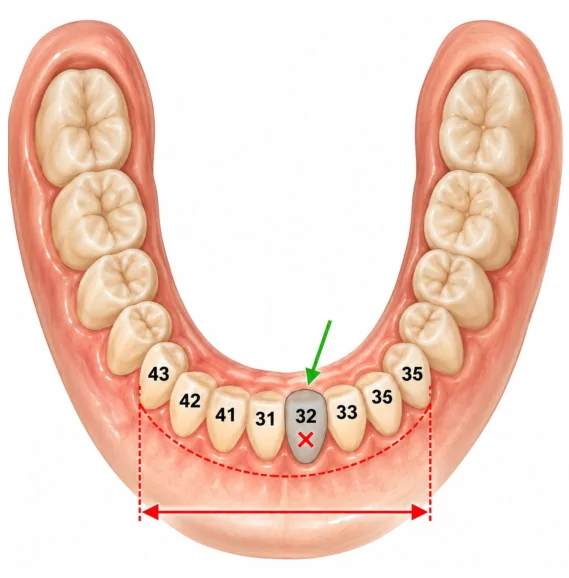
Scheme of the mandibular bone cyst, extending from FDI tooth 43 to FDI tooth 35. Tooth 32 showed no response to either the cold test or the electric pulp test (EPT), whereas all seven adjacent teeth (FDI 43, 42, 41, 31, 33, 34, and 35) exhibited normal physiological responses to both pulp sensibility tests (the green arrow indicates FDI tooth 32, identified as the site of origin of the lesion).

In early December 2022, a 71-year-old woman presented to the Specialized Hospital for Head and Neck Diseases, Akadémia Košice, n.o., with progressive swelling, pain, and deformity of the anterior mandible. Her medical history was significant for previous myocardial infarction, controlled arterial hypertension, and hypothyroidism. The patient was an active smoker, which was considered a potential risk factor for impaired bone healing. Given her medical history, associated comorbidities, the characteristics of the mandibular defect, and the planned surgical intervention, she was strongly advised to reduce cigarette consumption. Throughout follow-up, her underlying medical conditions and ongoing pharmacological treatment were regularly reviewed in consultation with her treating specialists. Clinical and radiographic examinations revealed an extensive radiolucent lesion involving the anterior mandible (Figure 1).

**Figure 2:**
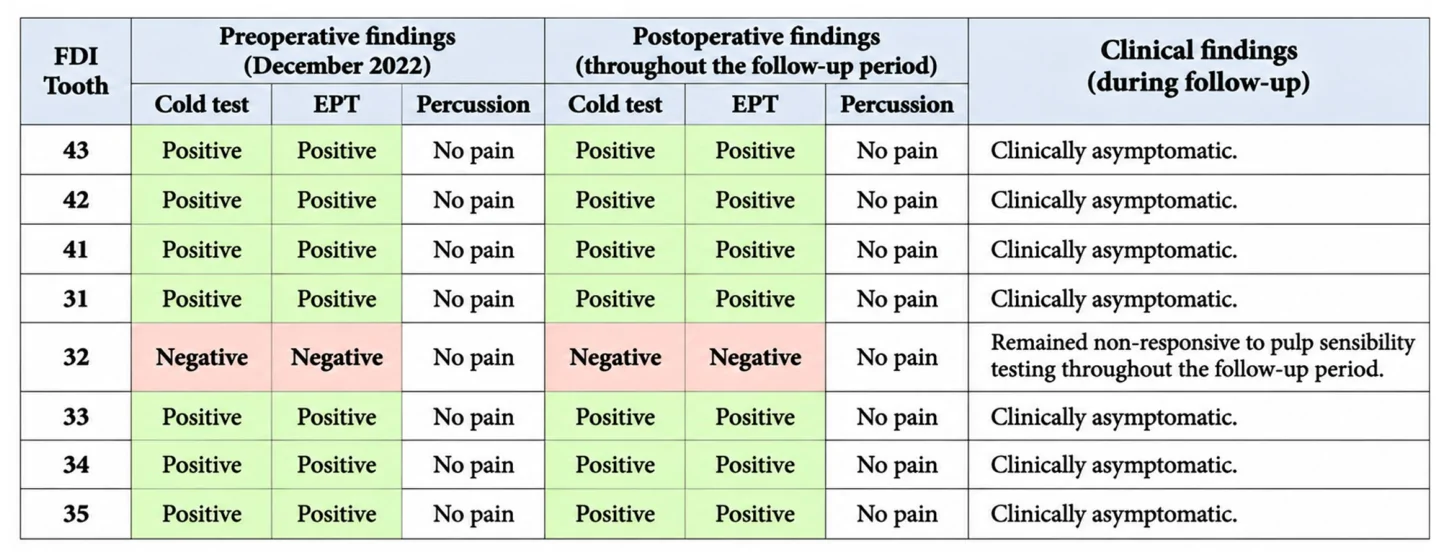
Preoperative and postoperative clinical findings and pulp sensibility test results for mandibular teeth (FDI 43, 42, 41, 31, 32, 33, 34, and 35) associated with the cystic lesion.

Preoperative pulp sensibility testing revealed positive responses to both cold testing and electric pulp testing (EPT) in seven adjacent teeth (FDI 43, 42, 41, 31, 33, 34, and 35). In contrast, tooth 32 exhibited no response to either test. The periodontal ligament spaces of all seven adjacent teeth were radiographically preserved. A tooth-specific summary of the preoperative and postoperative pulp sensibility findings is provided in Figure 2.

The cortical bone was largely preserved, although slight expansion of the buccal cortical plate was observed in the region of FDI teeth 32–33.

**Figure 3:**
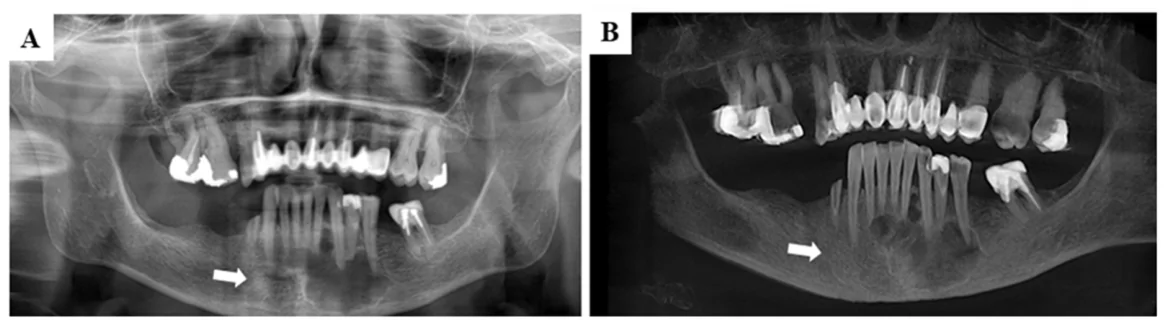
Initial preoperative panoramic radiographs (2D OPG). (**A**) Original panoramic radiograph demonstrating a well-defined radiolucent lesion of the anterior mandible extending from tooth FDI 43 to tooth FDI 35. (**B**) Contrast-enhanced image of the same radiograph highlighting the extent of the radiolucent lesion. The arrow indicates the location of the defect.

Preoperative imaging revealed a well-defined, unilocular radiolucent lesion measuring 27 × 35 × 6 mm (height × width × depth), extending from the right mandibular canine (FDI tooth 43) to the left mandibular second premolar (FDI tooth 35) (Figure 3A,B). Based on the combined clinical and radiographic findings, tooth 32 was identified as the most likely source of the lesion (Figure 1). The absence of responses to cold testing and electric pulp testing (EPT), together with associated periapical radiographic changes, suggested possible pulp necrosis and an endodontic origin of the lesion (Figure 10A). However, the negative pulp sensibility responses alone did not establish a definitive diagnosis of pulp necrosis. Tooth 32 remained non-responsive to both tests throughout postoperative follow-up. No endodontic treatment or other intervention involving this tooth was performed before, during, or after surgery. Based on the overall clinical and radiographic findings, a preliminary diagnosis of a radicular cyst involving the anterior mandible was established.

### 2.2. Surgical Procedure, Preparation and Application of the Calcium Phosphate Cement

The surgical procedure was performed on 22 December 2022. As part of the preoperative management, ANOPYRIN 100 mg (Zentiva, a.s., Bratislava, Slovakia) was temporarily discontinued five days before surgery to reduce the risk of excessive perioperative bleeding. Antibiotic therapy was initiated two days before surgery and consisted of AUGMENTIN 625 mg (GlaxoSmithKline Trading Services Limited, Dubline, Ireland), administered orally every 8 h, and ENTIZOL 250 mg (Zakłady Farmaceutyczne POLPHARMA S.A., Starogard Gdańsk, Poland), administered orally twice daily.

The surgical procedure was performed under local infiltration and regional conduction anesthesia supplemented with pharmacological sedation and analgesia. The medications administered included 4% SUPRACAINE (6 mL; Zentiva, k.s., Prague, Czech Republic), DOLSIN (1 mL; Pharma a.s., Roztoky, Czech Republic), and ATROPIN BBP (0.5 mg/mL; BB Pharma s.r.o., Martin, Slovakia).

Following preparation of the surgical field, a submarginal incision was made in the gingival mucosa between the mental foramina, with the flap extending toward the oral vestibule. The mucoperiosteal flap was carefully elevated using blunt dissection to minimize the risk of neurovascular injury and intraoperative bleeding. Subsequent exposure of the underlying bone revealed a defect in the buccal aspect of the mandible measuring 2.7 × 3.5 × 0.6 cm. The defect was associated with an extensive inflammatory lesion involving the anterior mandibular region, extending from FDI tooth 43 to FDI tooth 35.

Based on the preoperative clinical examination, negative pulp sensibility responses, and associated radiographic findings, tooth 32 was considered the probable source of the inflammatory lesion. The decision to retain tooth 32 without endodontic treatment was made by the operating maxillofacial surgeon following an individualized assessment of the clinical and radiographic findings and the overall surgical management strategy. Although tooth 32 showed no response to pulp sensibility testing, these findings alone were insufficient to establish a definitive diagnosis of pulp necrosis. Accordingly, the tooth was retained without endodontic intervention and remained under clinical and radiographic surveillance throughout the postoperative follow-up period.

A cystic lesion containing purulent material was identified at the defect site. Complete surgical enucleation of the cyst, including its contents, was subsequently performed by an experienced maxillofacial surgeon. The procedure was carried out using meticulous surgical technique to minimize contamination of the surrounding tissues. As the cyst was well encapsulated, it was removed without disruption of the cyst wall, thereby avoiding spillage of the purulent contents into the surrounding tissues.

Following cyst removal, the surgical cavity was irrigated with an antiseptic solution prepared by mixing 3% hydrogen peroxide (H_2_O_2_; VULM, a.s., Bratislava, Slovakia) and 0.2% povidone-iodine solution (Betadine; Mundipharma AG, Basel, Switzerland) in a 1:1 volume ratio, yielding nominal final concentrations of 1.5% H_2_O_2_ and 0.1% povidone-iodine, respectively. This irrigation protocol was selected based on the surgical team’s previous clinical experience with the use of these antiseptic agents in dental procedures. No clinically apparent adverse effects on the surrounding tissues were observed following the single intraoperative application in the present case.

**Figure 4:**
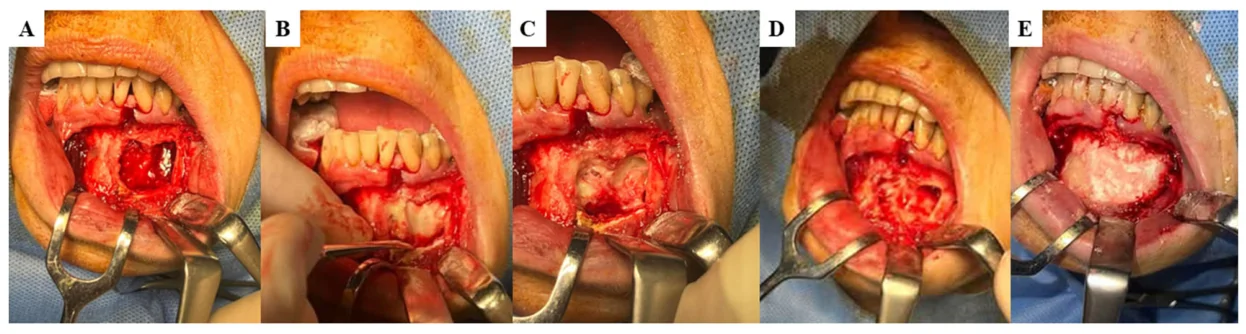
Intraoperative photographs illustrating the surgical procedure. (**A**) Removal of the buccal cortical bone, with the bone defect still covered by the periosteum. (**B**) Exposure of the pathological lesion with the cyst wall visible. (**C**) Dissection of the cyst from the surrounding tissues. (**D**) Complete enucleation of the cyst. (**E**) Filling of the bone defect with calcium phosphate biocement paste.

Following hemostasis, calcium phosphate cement (Biocement C) paste was prepared by mixing 5.67 g of cement powder with 2.835 mL of a 2% NaH_2_PO_4_ solution, corresponding to a powder-to-liquid ratio of 2 g/mL. The resulting paste was applied to the surgically selected portion of the mandibular bone defect using a 2 mL syringe (Figure 4A–E). The initial quantity of cement was prepared to ensure that sufficient material was available for reconstruction. However, approximately three-quarters of the prepared paste was implanted, as the region corresponding to FDI teeth 31, 32, and 41 was intentionally left unfilled based on the surgeon’s intraoperative clinical assessment. The proportion of paste implanted was estimated during the procedure; neither the exact volume of the prepared paste nor the volume actually implanted was directly measured. The surgical wound was subsequently closed with interrupted nonabsorbable sutures.

**Figure 5:**
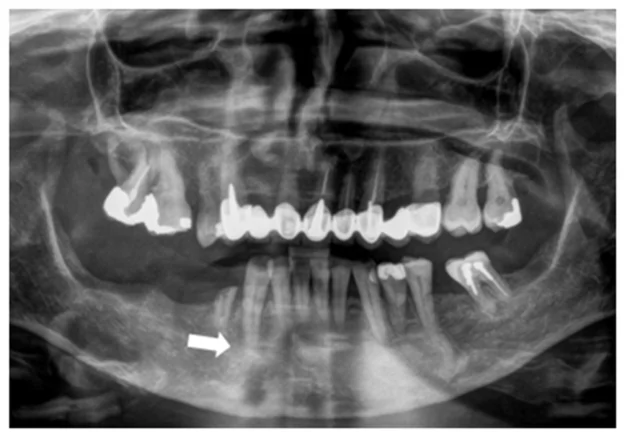
Immediate postoperative panoramic radiograph (2D OPG) demonstrating filling of the mandibular bone defect with calcium phosphate cement after enucleation of the radicular cyst. The arrow indicates the location of the defect.

Immediately after surgery, a two-dimensional panoramic radiographic examination (2D OPG) was performed to assess the placement and distribution of the calcium phosphate cement within the mandibular bone defect (Figure 5). The biomaterial occupied the surgically selected portion of the defect and was in contact with the surrounding bone. The region corresponding to FDI teeth 31, 32, and 41 was intentionally left unfilled based on the operating surgeon’s clinical judgment and intraoperative assessment. Given the relatively limited extent of the defect in this region and its anticipated capacity for spontaneous healing, additional reconstruction with Biocement C was considered unnecessary.

The excised cystic tissue was submitted for histopathological examination, while the purulent cystic contents were collected for microbiological culture and antimicrobial susceptibility testing.

Following surgery, the patient continued the preoperatively initiated antibiotic regimen for an additional 10 days. This consisted of AUGMENTIN 625 mg (GlaxoSmithKline Trading Services Limited, Dubline, Ireland), administered orally as one tablet every 8 h, and ENTIZOL 250 mg (Zakłady Farmaceutyczne POLPHARMA S.A., Starogard Gdańsk, Poland), administered orally as one tablet twice daily. ANOPYRIN 100 mg (Zentiva, a.s., Bratislava, Slovakia) was resumed five days after surgery, and the patient received standard postoperative care.

Postoperative instructions included temporary dietary modifications, local cooling of the surgical site, the use of analgesics as needed, and attendance at scheduled follow-up examinations. The postoperative course was uneventful, with no clinically apparent complications, and the patient remained under regular clinical and radiographic follow-up.

The surgical wound was monitored daily, and the patient continued the postoperative antibiotic regimen described above. The nonabsorbable sutures were removed 10 days after surgery. A follow-up examination was conducted two weeks postoperatively, followed by monthly clinical examinations throughout 2023. In 2024, two additional follow-up examinations were performed, with the final assessment taking place in August 2024. The follow-up schedule was established by the operating maxillofacial surgeon, who also supervised the patient’s clinical monitoring throughout the postoperative period.

At each follow-up examination, wound healing, the condition of the surrounding tissues, and the occurrence of potential postoperative complications were assessed. Pulp sensibility was also evaluated using cold testing and electric pulp testing (EPT) in eight teeth (FDI 43, 42, 41, 31, 32, 33, 34, and 35). Throughout the postoperative follow-up period, tooth 32 remained non-responsive to both tests, whereas the seven adjacent teeth (FDI 43, 42, 41, 31, 33, 34, and 35) consistently exhibited positive responses. During the follow-up period, the patient missed approximately three scheduled clinical appointments due to illness; these appointments were subsequently rescheduled.

Postoperative panoramic radiographs (OPG) were obtained at the monthly clinical follow-up examinations during 2023 and at the first follow-up examination in 2024, in accordance with the schedule established by the operating maxillofacial surgeon. At the final follow-up examination in August 2024, cone-beam computed tomography (CBCT) was performed to obtain a more detailed three-dimensional assessment of the mandibular defect and its radiographic healing characteristics.

The documented postoperative radiographic examinations included those performed on 9 January, 1 February, 23 March, and 5 April 2023. The panoramic radiographs presented in Figure 9A–D were selected from the available radiographic documentation to illustrate the postoperative follow-up.

**Figure 6:**
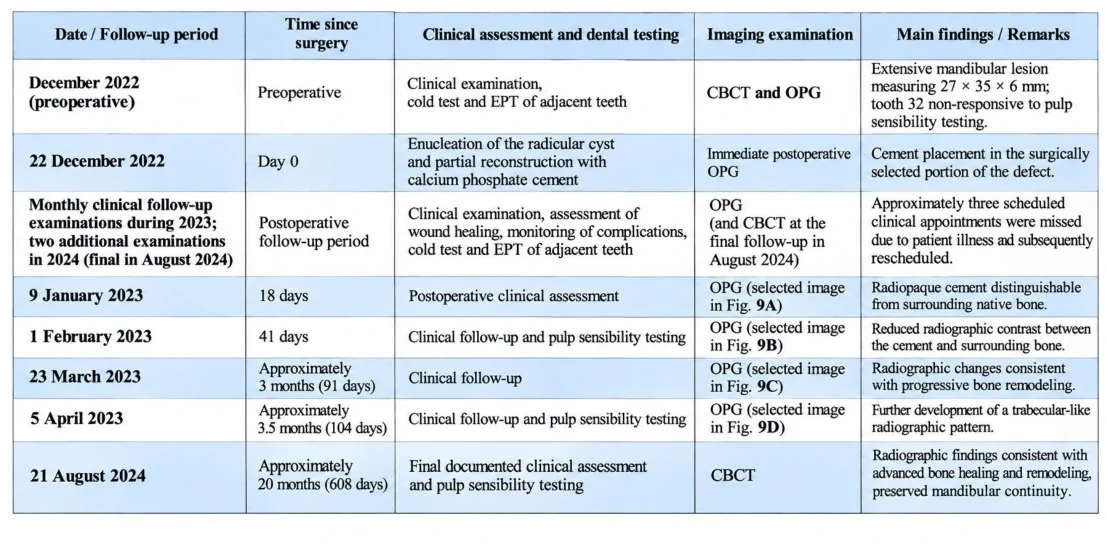
Chronological overview of postoperative clinical and radiographic follow-up.

Two cone-beam computed tomography (CBCT) examinations were performed: the first preoperatively in December 2022 and the second on 21 August 2024, as part of the final radiographic assessment. A chronological overview of the clinical and radiographic follow-up examinations, together with the corresponding documented findings, is presented in Figure 6.

### 2.3. Fabrication and Physicochemical Characterization of Calcium Phosphate Cement

The calcium phosphate cement (Biocement C) consisted of a powder mixture containing tetracalcium phosphate (TTCP) and nanomonetite. TTCP was synthesized by heating an equimolar mixture of CaCO_3_ and CaHPO_4_ at 1450 °C for 5 h, followed by milling in a planetary ball mill for 2 h. Nanomonetite was subsequently prepared by reaction milling of TTCP with orthophosphoric acid diluted in 90% ethanol for 30 min. The Ca/P molar ratio of the cement powder mixture was maintained at 1.67. Aqueous NaH_2_PO_4_ solutions at concentrations of 2% and 4% were used as setting liquids, with a powder-to-liquid ratio of 2 g/mL.

**Figure 7:**
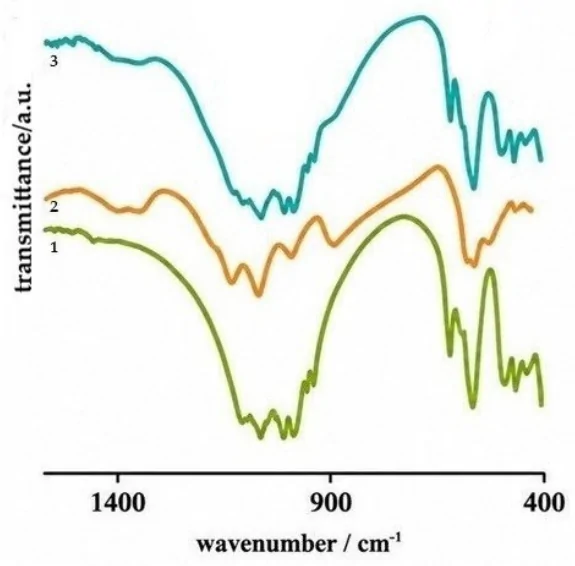
FTIR spectra demonstrating phase transformation into hydroxyapatite. FTIR spectra of synthesized TTCP (1), monetite (2), final mixture after reaction with orthophosphoric acid (3).

**Figure 8:**
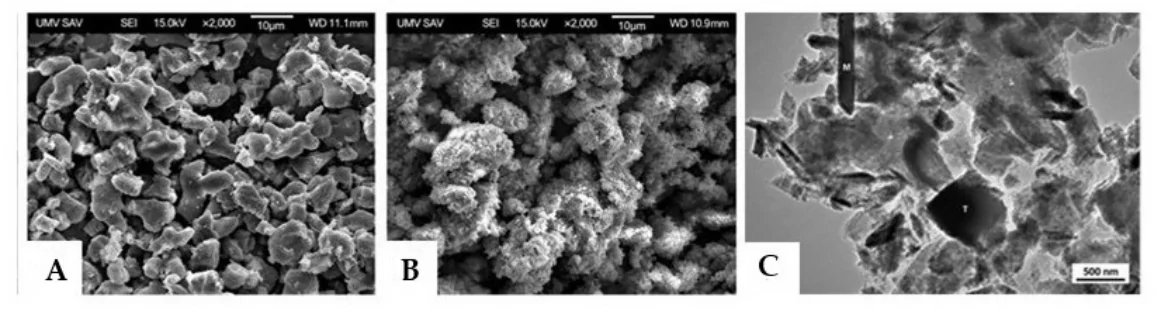
Morphology of TTCP particles in cement mixture before (**A**), and after reaction (**B**), with orthophosphoric acid solution observed by SEM micrographs showing details of particle morphologies in mixture after reaction (**C**).

The physicochemical properties of this cement formulation have been characterized in previously published studies [11,18]. X-ray diffraction (XRD) analysis confirmed the presence of TTCP and monetite in the initial powder mixture and its complete conversion to nanocrystalline hydroxyapatite after 24 h of hardening. Scanning and transmission electron microscopy (SEM/TEM) revealed a homogeneous distribution of nanomonetite particles, including nanoparticles approximately 10 nm in size that formed larger agglomerates (Figure 8C). Fourier-transform infrared spectroscopy (FTIR) identified characteristic phosphate vibrational bands and confirmed the formation of partially carbonated hydroxyapatite (Figure 7). The hardened cement exhibited a microporous microstructure consisting of needle-like hydroxyapatite nanocrystals and a compressive strength of 35 ± 6 MPa (Figure 8A,B). The reported setting times were approximately 9 min and 4 min when 2% and 4% NaH_2_PO_4_ solutions were used, respectively.

For clinical reconstruction, 5.67 g of Biocement C powder was mixed with 2.835 mL (2835 µL) of a 2% NaH_2_PO_4_ solution, corresponding to a powder-to-liquid (P/L) ratio of 2 g/mL. The mixing procedure took approximately 5 min, after which the prepared paste was immediately applied to the surgically selected portion of the mandibular defect. The reported quantities refer to the individual components used for cement preparation and should not be interpreted as the final volume of either the prepared or implanted paste [11,18]. Before clinical application, the TTCP powder component was sterilized by dry heat at 160 °C for 2 h in a hot-air sterilizer. The 2% NaH_2_PO_4_ setting solution was sterilized by filtration through a 0.2 µm membrane filter under aseptic conditions. As part of the quality-control procedure, a representative sample from the production batch was submitted for microbiological examination before clinical use. The cement was used for clinical application only after satisfactory microbiological examination results had been obtained and documented.

### 2.4. Radiographic Evaluation

Radiographic evaluation included digital panoramic radiography (2D OPG) and cone-beam computed tomography (CBCT). All examinations were performed using an ORTHOPHOS XG3D imaging system (Sirona Dental Systems GmbH, Bensheim, Germany). Panoramic radiography was used for the initial assessment and postoperative follow-up, whereas CBCT was employed for three-dimensional evaluation of the lesion, preoperative surgical planning, and long-term assessment of radiographic changes associated with bone healing and remodeling.

Both the preoperative and final follow-up CBCT examinations were performed using a tube voltage of 85 kV, a tube current of 13 mA, and an exposure time of 5 s, with a recorded dose–area product (DAP) of 627 mGy·cm^2^. The XG3D 1.5 acquisition software was used for both examinations.

Radiographic follow-up was based exclusively on qualitative assessment of the acquired images. The evaluation focused on changes in radiopacity, the development of a trabecular-like radiographic pattern, preservation of mandibular cortical continuity, and the presence or absence of pathological changes or lesion recurrence. No serial quantitative measurements of defect dimensions, bone volume, or biomaterial degradation were performed.

All OPG and CBCT images were evaluated by the operating maxillofacial surgeon, who had extensive clinical experience in maxillofacial surgery. The observed radiographic changes were therefore interpreted descriptively and were not considered quantitative evidence of bone regeneration.

### 2.5. Postoperative Management and Follow-Up

Postoperative healing was assessed through regular clinical and radiographic examinations throughout the follow-up period. The postoperative course was uneventful, with no evidence of wound dehiscence, local infection, inflammatory complications, or lesion recurrence. No complications attributable to the patient’s underlying medical conditions or long-term pharmacological treatment were identified during follow-up. All seven adjacent teeth (FDI 43, 42, 41, 31, 33, 34, and 35) remained clinically asymptomatic and consistently demonstrated positive responses to pulp sensibility testing. In contrast, tooth 32 remained non-responsive to both cold testing and electric pulp testing (EPT), consistent with the preoperative findings. Potential postoperative complications were assessed during routine clinical examinations and documented in the patient’s medical records. Clinical evaluations included assessment of wound healing, local inflammatory changes, pain, functional complaints, and potential adverse events. Patient-reported symptoms and functional status were evaluated through routine clinical interviews without standardized pain scales or validated quality-of-life questionnaires. Accordingly, the reported outcomes reflect clinical observations and the patient’s subjective experiences rather than quantitatively measured improvements in quality of life.

**Figure 9:**
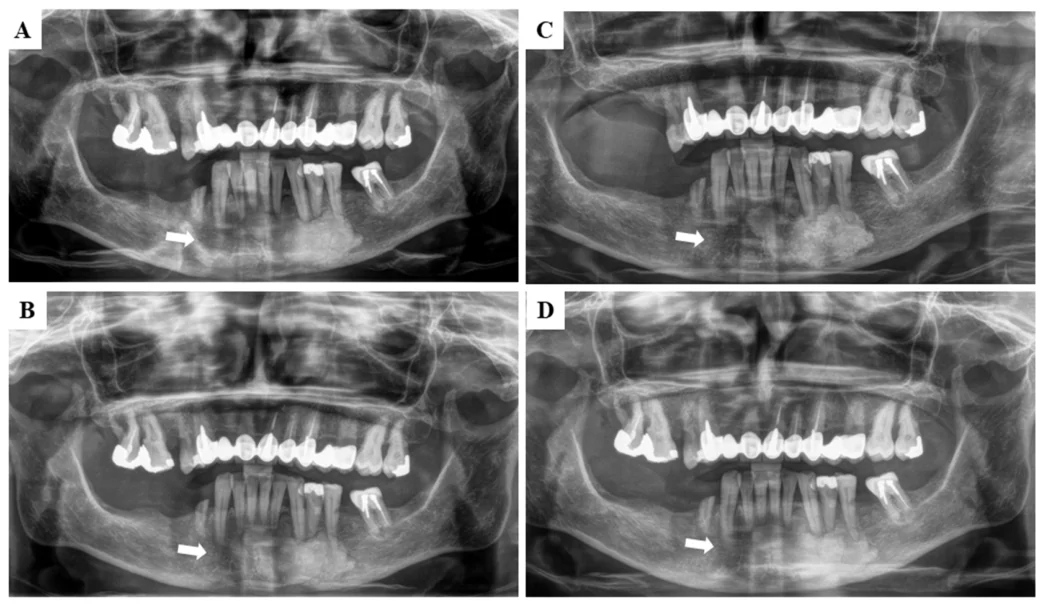
Serial postoperative panoramic radiographs (2D OPG) obtained during follow-up after reconstruction of the mandibular bone defect with calcium phosphate cement, showing radiographic changes consistent with progressive bone healing and remodeling. (**A**) 9 January 2023. (**B**) 1 February 2023. (**C**) 23 March 2023. (**D**) 5 April 2023. The arrow indicates the location of the bone defect.

The surgical procedure was performed on 22 December 2022, and the first follow-up panoramic radiographic examination (OPG) was conducted on 9 January 2023, approximately three weeks postoperatively. The panoramic radiograph demonstrated the presence of calcium phosphate cement within the surgically selected portion of the mandibular bone defect. The implanted biomaterial exhibited relatively homogeneous high radiopacity, clearly distinguishable from the surrounding native bone. A well-defined radiographic interface was visible between the biomaterial and the adjacent bone (Figure 9A).

At the subsequent follow-up examination on 1 February 2023, a gradual reduction in radiographic contrast between the implanted cement and the surrounding bone was observed. The periodontal ligament spaces of all adjacent teeth remained preserved without evidence of root resorption or periapical pathology. Positive responses to both cold testing and electric pulp testing (EPT) were confirmed in all seven adjacent teeth (FDI 43, 42, 41, 31, 33, 34, and 35), whereas tooth 32 remained non-responsive to both tests (Figure 9B).

At the subsequent follow-up examination on 1 February 2023, a reduction in radiographic contrast between the implanted cement and the surrounding native bone was observed. The periodontal ligament spaces of the seven adjacent teeth (FDI 43, 42, 41, 31, 33, 34, and 35) remained preserved, with no radiographic evidence of root resorption or periapical pathology in these teeth. All seven teeth continued to exhibit positive responses to both cold testing and electric pulp testing (EPT), whereas tooth 32 remained non-responsive to both tests (Figure 9B).

At the follow-up examination on 23 March 2023, radiographic changes consistent with progressive remodeling were observed in the reconstructed region. The central portion of the original defect exhibited a transition from an initially homogeneous radiopaque appearance to a trabecular-like radiographic pattern. Concurrently, the radiographic boundary between the reconstructed region and the surrounding native bone became less distinct (Figure 9C).

At the follow-up examination on 5 April 2023, further radiographic changes were observed, including the development of a trabecular-like radiographic pattern and focal areas of increased radiodensity within the reconstructed region. All seven adjacent teeth (FDI 43, 42, 41, 31, 33, 34, and 35) continued to demonstrate positive responses to both cold testing and electric pulp testing (EPT), whereas tooth 32 remained non-responsive to both tests (Figure 9D). Clinical assessment of the affected teeth included cold testing and EPT to evaluate pulp sensibility, as well as percussion testing to assess the surrounding periodontal and periapical tissues. These examinations were performed during postoperative follow-up visits to monitor the clinical status of the teeth.

**Figure 10:**
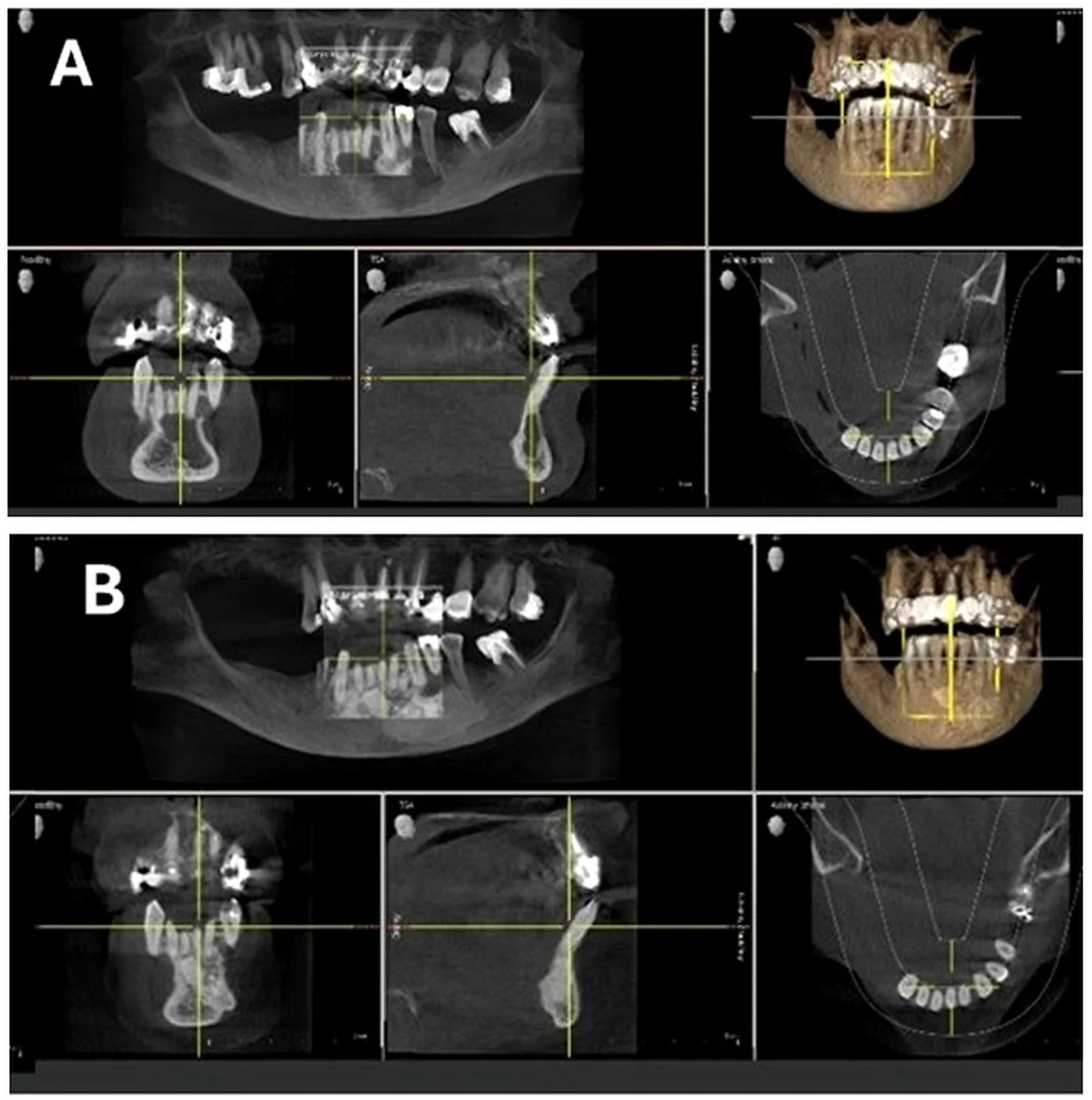
Comparative cone-beam computed tomography (CBCT) images of the mandible showing (**A**) the preoperative appearance of the mandibular bone defect (December 2022) and (**B**) the findings at the final follow-up examination (August 2024) after reconstruction with calcium phosphate cement. Both panels include multiplanar CBCT views and three-dimensional reconstructions, enabling qualitative assessment of radiographic changes in the affected mandibular region over time.

The final CBCT examination, performed on 21 August 2024, revealed radiographic changes consistent with progressive healing and remodeling of the reconstructed mandibular region, with preservation of mandibular continuity. The reconstructed region exhibited a trabecular-like radiographic architecture resembling that of the surrounding native mandibular bone. Subtle differences in radiopacity remained visible along the peripheral margins of the original defect. Cortical continuity was preserved, and no new pathological changes were identified in the periodontal ligament spaces or periapical regions of the seven adjacent teeth (FDI 43, 42, 41, 31, 33, 34, and 35). A distinct radiopaque line extending from the region of FDI tooth 35 was identifiable on CBCT, corresponding to the original mandibular cortical boundary. A smooth radiographic transition between the surrounding native bone and the reconstructed region was also observed (Figure 10B). However, the radiographic appearance of residual calcium phosphate cement may overlap with that of newly formed mineralized bone. Therefore, the extent of cement degradation and replacement by newly formed bone could not be definitively established from CBCT findings alone.

### 2.6. Histopathological and Microbiological Findings

**Figure 11:**
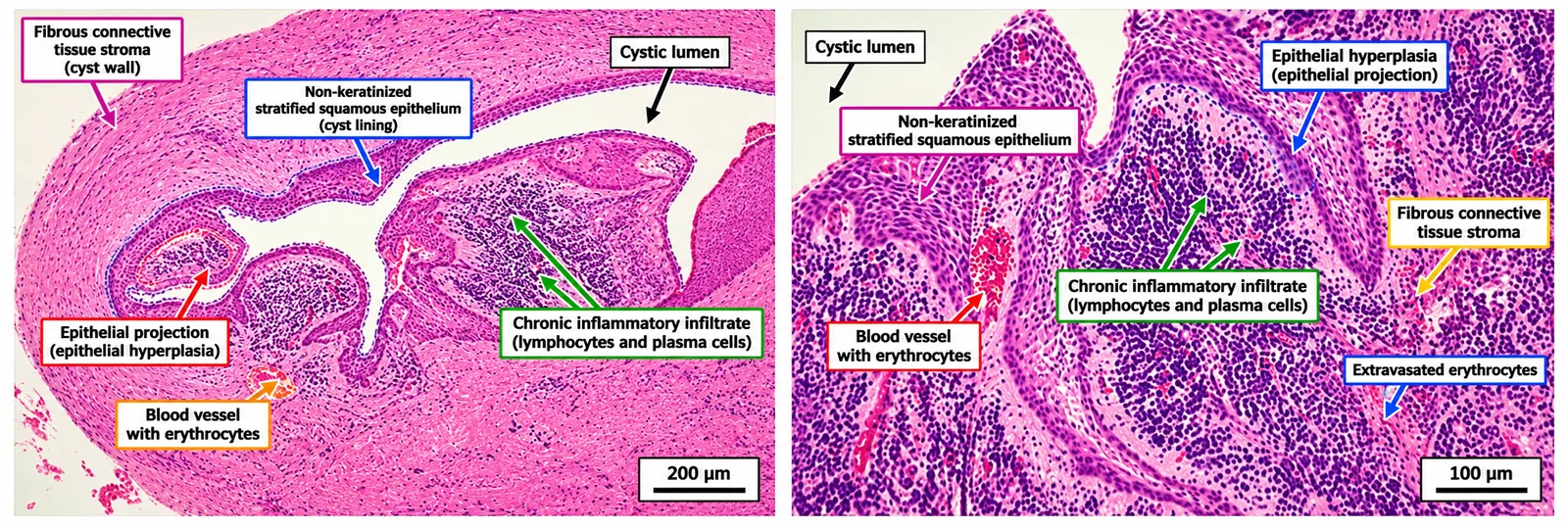
Histopathological features of the radicular cyst. Representative hematoxylin and eosin (H&E)-stained sections showing a cyst wall lined by non-keratinized stratified squamous epithelium with reactive epithelial hyperplasia and irregular epithelial projections. The underlying fibrous connective tissue exhibits chronic inflammatory cell infiltration, predominantly consisting of mononuclear cells, together with small blood vessels and focal hemorrhagic changes.

Histopathological examination revealed a benign radicular cyst lined by non-keratinized stratified squamous epithelium, without evidence of epithelial dysplasia. The cyst wall exhibited focal chronic nonspecific inflammatory and reparative changes, with no evidence of neoplastic transformation or other significant pathological alterations. These findings were consistent with the clinical diagnosis of a radicular cyst (*cystis radicularis mandibulae*) in the anterior mandible, associated with the root of FDI tooth 32 (Figure 11). Microbiological culture of the purulent cystic contents revealed the presence of *Staphylococcus* spp. and *Streptococcus* spp. Antimicrobial susceptibility testing demonstrated that the isolated microorganisms were susceptible to amoxicillin/clavulanic acid (Augmentin).

## 3. Discussion

Extensive mandibular bone defects, particularly those resulting from the enucleation of large odontogenic cysts, remain a significant clinical challenge in oral and maxillofacial surgery. Defect size can substantially influence the healing process, mandibular biomechanical stability, and the predictability of bone regeneration [24]. Consequently, extensive defects may require reconstruction using bone grafts or synthetic biomaterials to support bone regeneration and preserve the structural integrity of the mandible [25,26].

Calcium phosphate cements (CPCs) have emerged as promising biomaterials for bone defect reconstruction because of their chemical similarity to the mineral phase of human bone [25]. Their generally favorable biocompatibility and osteoconductive properties, together with their ability to conform to irregularly shaped bone defects and harden in situ, support their potential application in bone reconstruction [11,17,18,27,28]. These characteristics may facilitate close adaptation of the cement to the surrounding bone tissue and provide a suitable environment for subsequent bone remodeling. CPCs may therefore represent an alternative to autologous bone grafting in selected clinical situations. Nevertheless, autologous bone grafting remains the gold standard for the reconstruction of many bone defects because of its combined osteogenic, osteoinductive, and osteoconductive properties [29]. Its limitations include donor-site morbidity, limited graft availability, prolonged operative time, and potential postoperative complications [30,31,32,33].

Although substantial progress has been made in experimental research on calcium phosphate cements, clinical evidence regarding their use in the reconstruction of extensive mandibular bone defects remains limited [31,34]. Recent systematic reviews indicate that the available evidence predominantly concerns preclinical models or smaller alveolar defects, whereas clinical studies involving extensive mandibular defects and long-term follow-up remain scarce [25,26].

The present case report contributes to the limited clinical literature on the application of calcium phosphate cements in craniofacial bone reconstruction [35]. Long-term clinical and CBCT follow-up demonstrated radiographic changes consistent with progressive remodeling of the reconstructed mandibular region. Throughout the follow-up period, all seven adjacent teeth (FDI 43, 42, 41, 31, 33, 34, and 35) maintained positive responses to pulp sensibility testing, whereas tooth 32 remained non-responsive to both cold and electric pulp testing. No local postoperative complications were observed.

Experimental studies have shown that certain calcium phosphate cement formulations can support vascularization and new bone formation, with some formulations exhibiting gradual degradation and replacement by newly formed bone, without a pronounced inflammatory response [16,27]. Furthermore, their injectability and moldability facilitate adaptation to complex bone defect geometries, highlighting their potential advantages in mandibular reconstruction following the enucleation of extensive odontogenic lesions [27].

These properties further support the potential application of calcium phosphate cements in the reconstruction of extensive bone defects, particularly in selected patients for whom avoiding donor-site morbidity associated with autologous bone harvesting may be clinically beneficial [8,24,28,36].

In the present case, long-term clinical and radiographic follow-up provided valuable information on the postoperative evolution of the reconstructed mandibular defect. Serial radiographic examinations, including the final CBCT assessment, revealed changes in radiopacity and the development of a trabecular-like radiographic pattern, together with preservation of mandibular continuity and no radiographic evidence of lesion recurrence. These findings are consistent with a favorable clinical and radiographic outcome following calcium phosphate cement implantation. However, the extent of new bone formation and cement degradation could not be definitively established from the available imaging findings. Experimental studies have reported gradual degradation of certain calcium phosphate cement formulations and their replacement by newly formed bone, accompanied by a favorable biological response of the surrounding tissues [27,36].

Furthermore, the diagnostic value of CBCT in assessing periapical conditions and treatment outcomes has been highlighted in a systematic review and meta-analysis by Martins et al. [37]. Although that study focused on endodontic treatment outcomes rather than bone regeneration following cyst enucleation, its findings support the clinical relevance of CBCT for evaluating periapical structures.

Clinical studies investigating spontaneous bone healing following the enucleation of jaw cysts have indicated that healing outcomes may be less predictable in extensive defects and may be influenced by the initial lesion size [38,39]. These findings highlight the importance of an individualized approach when considering bone substitutes for the reconstruction of large jaw defects, particularly in cases where spontaneous healing may not achieve the desired clinical outcome.

One of the most important clinical findings in the present case was the preservation of positive responses to pulp sensibility testing in all seven teeth adjacent to the bone defect throughout the follow-up period. Large radicular cysts may cause root displacement and extensive alveolar bone destruction, potentially affecting the pulpal status of adjacent teeth and increasing the likelihood of subsequent endodontic treatment or tooth extraction [40]. Nevertheless, available clinical evidence suggests that careful surgical management, including atraumatic surgical techniques and preservation of the surrounding neurovascular structures, may contribute to maintaining pulp sensibility in adjacent teeth [41].

In a prospective three-year clinical study, Niu et al. [41] reported that positive responses to pulp sensibility testing were preserved in the majority of teeth located within regions affected by jaw cysts, whereas only a small proportion required subsequent endodontic treatment. The findings of the present case are consistent with these observations, as all seven adjacent teeth (FDI 43, 42, 41, 31, 33, 34, and 35) maintained positive responses to pulp sensibility testing throughout the follow-up period without requiring endodontic intervention. These observations emphasize the importance of a minimally traumatic surgical approach and support consideration of a conservative treatment strategy in which prophylactic endodontic treatment of adjacent teeth is avoided unless clinically or radiographically indicated [42].

The management of tooth 32 represents an important clinical consideration in the present case. The absence of responses to cold testing and electric pulp testing, together with the associated periapical radiographic findings, suggested possible pulp necrosis and an endodontic origin of the lesion. However, these findings alone did not definitively establish the pulpal status of the tooth. The decision to retain tooth 32 without endodontic treatment was based on an individualized clinical assessment and the overall surgical management strategy of the operating maxillofacial surgeon. Complete cyst enucleation was performed, followed by regular clinical and radiographic monitoring. Tooth 32 remained non-responsive to pulp sensibility testing throughout the follow-up period. Importantly, the absence of endodontic treatment represents a limitation of the present case, as a persistent intraradicular source of infection cannot be definitively excluded. Therefore, the favorable postoperative healing observed in this patient should not be interpreted as evidence that endodontic treatment is unnecessary in comparable clinical situations.

Another important aspect of the present case was the long-term radiographic follow-up, including the final CBCT examination, which enabled three-dimensional assessment of the reconstructed mandibular region. Compared with conventional panoramic radiography, CBCT provides more detailed visualization of the bone defect and surrounding cortical and trabecular structures [43]. In the present case, serial radiographic examinations revealed changes in radiopacity, the development of a trabecular-like radiographic pattern, and preservation of mandibular continuity, without radiographic evidence of lesion recurrence or pathological bone resorption in the surrounding region. These findings are consistent with progressive bone healing and remodeling. However, the radiographic appearance of residual calcium phosphate cement may overlap with that of newly formed mineralized bone, making reliable differentiation difficult. Consequently, the extent of cement degradation and its replacement by newly formed bone cannot be definitively established from imaging alone and would require histological confirmation. An additional limitation of this case report is the absence of serial quantitative radiographic measurements, as the assessment of bone healing and remodeling was based exclusively on qualitative imaging findings.

The findings of the present case can also be considered in the context of the retrospective cohort study by La Rosa et al. [38], which examined bone healing following jaw cyst enucleation using CBCT and identified initial lesion size as a factor influencing spontaneous bone regeneration. Given the extent of the mandibular defect in the present case, the favorable long-term clinical and radiographic findings provide additional clinical experience regarding the potential application of calcium phosphate cement in the reconstruction of extensive mandibular bone defects.

The lesion resulted in an extensive cavitary bone defect with preserved mandibular continuity, rather than a segmental defect involving disruption of mandibular continuity.

Considering the extent of the mandibular bone defect, the patient’s age, and associated comorbidities, spontaneous bone healing following cyst enucleation alone was considered insufficiently predictable to ensure a satisfactory reconstructive outcome. The decision to use calcium phosphate cement was based on these clinical considerations and the extensive clinical experience of the operating maxillofacial surgeon. Alternative reconstructive approaches were considered less advantageous in this particular clinical situation rather than formally contraindicated. The cement was selected to provide structural support and facilitate bone healing. Although the favorable long-term clinical and radiographic findings support the clinical rationale for the selected treatment approach, the specific contribution of the cement to the observed healing process cannot be conclusively established from a single case without a control group. The intentionally unfilled region was monitored descriptively and did not constitute an independent control site. No quantitative comparison between the cement-filled and unfilled regions was performed; therefore, these observations do not permit conclusions regarding the comparative effectiveness of calcium phosphate cement.

The present case report documents a favorable clinical and radiographic outcome following the reconstruction of an extensive mandibular bone defect with calcium phosphate cement after radicular cyst enucleation. Long-term follow-up revealed radiographic changes consistent with progressive bone healing and remodeling, preservation of mandibular continuity, and maintained positive responses to pulp sensibility testing in all seven adjacent teeth. No clinical or radiographic evidence of cyst recurrence or local postoperative complications was observed during the follow-up period, and no additional surgical or endodontic interventions were performed. Although these findings indicate a favorable outcome in this individual patient, they do not establish the specific contribution or comparative effectiveness of the implanted cement. Nevertheless, this case provides additional clinical experience supporting further investigation of calcium phosphate cement as a potential reconstructive option for extensive mandibular bone defects.

## 4. Conclusions

This case report describes a favorable clinical and radiographic outcome following the application of calcium phosphate cement for the reconstruction of a large mandibular bone defect after radicular cyst enucleation. The postoperative course was uncomplicated, and long-term clinical and radiographic follow-up demonstrated maintained positive responses to pulp sensibility testing in the seven adjacent teeth, preservation of mandibular continuity, and radiographic changes consistent with progressive bone remodeling.

Although these findings suggest that calcium phosphate cement may represent a promising reconstructive option in carefully selected patients, the evidence is limited to a single clinical case and does not establish its comparative efficacy or general applicability. Furthermore, the absence of quantitative radiographic analysis and histological confirmation precludes definitive conclusions regarding the extent of cement degradation, new bone formation, and the specific contribution of the implanted material to the observed healing process.

Further independent clinical studies involving larger patient populations, standardized quantitative assessments, and long-term follow-up are needed to evaluate the safety, clinical effectiveness, and appropriate indications for the use of calcium phosphate cements in mandibular bone defect reconstruction.

## Data Availability

Data is contained within this article.
